# Increased Erythrocyte Sedimentation Rate in Dogs: Frequency in Routine Clinical Practice and Association with Hematological Changes

**DOI:** 10.3390/ani14101409

**Published:** 2024-05-08

**Authors:** Saverio Paltrinieri, Roberta Ferrari, Donatella Scavone, Carolina Pieroni, Daniela Diamanti, Filippo Tagliasacchi

**Affiliations:** 1 Department of Veterinary Medicine and Animal Sciences, University of Milan, 26900 Lodi, Italy; roberta.ferrari@unimi.it (R.F.); donatella.scavone@guest.unimi.it (D.S.); filippo.tagliasacchi@unimi.it (F.T.); 2 DIESSE Diagnostica Senese SpA, 53035 Monteriggioni, Italy; carolinapieroni@diesse.it (C.P.); danieladiamanti@diesse.it (D.D.)

**Keywords:** acute phase response, anemia, canine, clinical pathology, complete cell blood count, ESR

## Abstract

**Simple Summary:**

The erythrocyte sedimentation rate (ESR) is a laboratory marker that may be used to evaluate animal welfare. The scientific literature has documented its increase in infectious and inflammatory diseases. However, there is no information on the frequency of an increased ESR in veterinary clinical practice, as well as on the possible association with different conditions in the canine patients. To fill this gap, the ESR was measured using the automated instrument MINI-PET (DIESSE Diagnostica Senese) and compared with other hematological parameters, using anticoagulated blood from dogs submitted to the clinical pathology laboratory of the veterinary teaching hospital of the University of Milan. Samples were divided according to the clinical presentation in a control group and in groups with other diseases or conditions known to be potentially characterized by an increased ESR. The highest ESR values were found in samples from dogs with chronic kidney disease and acute/subacute inflammation, followed by those from dogs with mild chronic disorders, severe/acute diseases, tumors (including dogs undergoing chemotherapy), and urinary disorders. In these conditions, the ESR increased in dogs with low hematocrit or high leukocyte counts, suggesting that anemia and acute phase reaction, even if not associated with clinically evident inflammation, may increase the ESR.

**Abstract:**

(1) Background: the erythrocyte sedimentation rate (ESR) has been reported to increase in some infectious or inflammatory diseases in dogs, but no information on the frequency of increases in a routine clinical setting exists. The aim of this study was to assess the frequency of an increased ESR in dogs and to investigate its possible association with hematologic changes; (2) Methods: A total of 295 EDTA blood samples were randomly selected from the routine caseload of the Veterinary Teaching Hospital. Samples were grouped in controls and in pathologic groups based on the clinical presentation. A routine hemogram was performed, then the ESR was measured using the instrument MINI-PET; (3) Results: compared with controls, the ESR was significantly higher in all the pathologic groups, except for the hematological disorders group. The highest ESR was found in samples from dogs with chronic kidney disease or inflammation, followed by those from dogs with mild chronic disorders, severe/acute diseases, tumors and urinary disorders. The ESR negatively correlated with hematocrit and positively with neutrophil counts. (4) Conclusions: The ESR increases more frequently in dogs with clinically evident inflammation or CKD, but also in several other conditions, likely as a consequence of anemia and acute phase response.

## 1. Introduction

The erythrocyte sedimentation rate (ESR) is widely used in human medicine as a marker of disease, prognosis, and follow-up, with a focus on inflammation [1]. The Westergren method was adopted by the International Council of Standardization in Hematology (ICSH) [2,3] as the reference method for ESR measurement. In this technique, fresh blood diluted in a 4:1 ratio, with sodium citrate as an anticoagulant, is aspirated into a fixed-diameter graduated pipette held vertically, and the ESR value is read after 60 min and expressed in millimeters/hour [4]. This marker is influenced by various biological factors, like sex, age [5], pregnancy, as well as technical factors such as temperature [6]. Gender and age are key determinants of the ESR, with females generally having a higher baseline ESR compared to males, and advancing age correlating with an increase in ESR [7,8]. Similar findings have been observed also in horses and dogs [8,9,10]. The sedimentation of red blood cells is faster in patients with acute inflammation and, therefore, ESR represents a non-specific marker for inflammatory disorders [11,12]. During inflammation, negatively charged sialic acid residues on red blood cells are covered by positively charged acute phase proteins, such as fibrinogen or immunoglobulins, inducing the formation of rouleaux and increasing the sedimentation rate [1,13,14].

The ESR has been quantified mostly in dogs infected with different pathogens and the increased ESR level is likely associated with an acute phase response (APR). Dogs with Canine leishmaniosis (CanL) have been reported to have the highest ESR level, with most of them (92%) presenting an increased ESR compared to exposed and healthy dogs [15]. Elevated ESR and C reactive protein (CRP) levels were found in dogs naturally infected with *Ehrlichia canis*, which presented mild to moderate inflammation [16]. In dogs naturally infected with *Babesia canis*, the ESR has been studied as an indicator of treatment success. Although it is a sensitive marker, with a tendency to decrease in treated dogs, the impact of anemia on its measurement must be considered when interpreting the results [17].

In the last few years, additional studies have been carried out on the ESR application as an important marker in animals’ inflammatory diseases [18,19], as well as in canine osteoarthritis [20] and in chronic kidney disease (CKD) [21]. However, in veterinary medicine, the ESR has been used in the past, but acute phase proteins are nowadays recommended when investigating inflammation [22,23,24,25] because conventional methods to measure ESR are time-consuming.

The recently commercialized automated patient-side instrument MINI-PET allows for the continuous loading of blood samples and enables rapid ESR measurement. This instrument analyzes blood samples in a shorter time compared with the manual method, using the same analytical principle. The reference limit of MINI-PET has already been established in dogs (10 mm/h) [19,26]. However, there is no information to date on the frequency of occurrence of an increased ESR in veterinary clinical practice, as well as on its possible association with diseases or clinical abnormalities in the dog, other than those previously mentioned. 

The purpose of this study was to evaluate the frequency of changes in the ESR in a case series of canine patients routinely examined in the clinical practice of a Veterinary Teaching Hospital, determining the clinical conditions that modify the ESR most frequently, and the possible association of this increase with other hematological changes. 

## 2. Materials and Methods

### 2.1. Caseload and Sample Processing

During the study period (April–August 2022), blood samples were randomly selected from those routinely sent to the clinical pathology laboratory of the Veterinary Teaching Hospital (VTH) of the University of Milan. All the samples were collected from the cephalic or the saphenous vein during diagnostic or monitoring procedures. At admission, the owners signed an informed consent form to authorize the use of leftover samples for research purposes. Therefore, according to the regulation of our Institution (decision 2/2016 of the Ethical Committee of the University of Milan), formal approval from the Ethical Committee was not required.

Since the aim of the study was to assess the conditions in which the ESR increases more frequently in clinical practice, the samples were included in the study regardless of the presence of a given disease, or of previous treatments. Similarly, multiple samples from the same dog at follow-up visits could have been included. The samples were grouped as specified below based on clinical information and the results of routine hematologic and biochemical tests, as well as on any other diagnostic procedures set by the referring veterinarian. 

Immediately after collection, the blood (1 mL) was placed in commercially available tubes with EDTA (Aptaca Spa, Canelli, AT, Italy). Tubes were filled to their maximum capacity and transported to the clinical pathology laboratory. A routine hemogram was performed using a laser-based cell counter (Sysmex XN, Sysmex, Kobe, Japan), followed by microscopic analysis of May Grunwald Giemsa-stained blood smears, to verify the differential leukocyte count and any possible morphologic change useful for diagnostic purposes. Among the data generated by the cell blood count, hematocrit, total leukocyte count, and neutrophil count were recorded for each sample to assess possible associations between an increased ESR and hematologic alterations such as anemia and leukocytosis.

Right after the hematologic analysis, the ESR was measured with the automated MINI-PET instrument (DIESSE Diagnostica Senese S.p.A., Monteriggioni, SI, Italy), based on a modified Westergren method. Following the manufacturer’s instructions, samples were gently mixed through complete inversion 10 times before being introduced into the instrument. Being calibrated to the reported dog reference times, its optical system measured the height of the plasma column formed after 14 min. Before each working session the instrument performs an autocalibration of its optical system. The coefficient of variation (CV) determined in intra-assay precision tests on ESR quality control material has been reported to be 9.8% for an ESR of 8 mm/h and 2.7% for an ESR of 12 mm/h [27]. 

### 2.2. Statistical Analysis

Based on clinical and laboratory data, samples were included in one of the following groups:

- Controls: samples from dogs that were clinically healthy at the time of collection and had no abnormalities in hematologic and biochemical analysis. These samples were collected as part of periodic health screenings, before elective surgeries (e.g., ovariectomy, orchiectomy) or diagnostic investigations (radiographs, ultrasound), or for monitoring patients in complete remission or those considered clinically cured. This group has been included in the study as a “negative” control, since changes in ESR values in this group were not expected.
- Mild chronic disorders: samples from dogs that, at the time of collection, had mild clinical signs or minimal laboratory changes associated with metabolic, degenerative, or chronic inflammatory conditions, mostly localized to a single organ or system. In this group, changes in the ESR were expected to be unlikely, either because some of the conditions included in this group do not have an inflammatory origin, or because the acute phase response associated with inflammation, if present, tends to become mild over time.
- Severe/acute diseases: samples from dogs that at the time of sampling had an acute clinical presentation or severe clinical conditions, likely associated with systemic involvement. This group may be of particular interest, because it included clinical events that may or may not have an inflammatory basis. For example, in some cases the acute clinical presentation may have led to sampling before the full development of inflammation (e.g., trauma or foreign bodies) or may have induced a stress response, with the production of cytokines that mimic an inflammatory pathogenesis. Additionally, in some of the acute diseases included in this group, the Ht is likely low, and this may influence the ESR, since a well-known inverse relationship between Ht and the ESR exists [23,28].
- Tumors: samples from dogs with different types of neoplasia, mainly lymphoproliferative disorders and mast cell tumors, collected either at first presentation or during chemotherapy. These samples, despite being collected from dogs affected by a chronic disorder, have been considered separately because they were particularly numerous and relatively homogeneous, and because it is known that the ESR may be increased in oncologic patients [29].
- Chronic Kidney Disease (CKD): samples from dogs that, at the time of collection, showed clinical and laboratory changes consistent with CKD in stage 2 of the International Renal Interest Society (IRIS) classification (http://www.iris-kidney.com, accessed on 1 March 2024) and/or a clinical presentation characterized by systemic symptoms referable to uremia. Dogs in the CKD group had a urine specific gravity (USG) < 1025, a documented history of azotemic CKD for more than 3 months, and no overt signs of uremia such as vomiting, anorexia, weakness, or uremic ulcers [30]. These samples, despite being affected by a chronic disorder, were considered as a separate group because they were relatively homogeneous in terms of pathogenesis and more easily clinically characterized.
- Urinary disorders: samples from dogs with acute symptoms related to inflammatory or obstructive disorders of the urinary tract. These samples, despite the acute presentation and an inflammatory pathogenesis, have been considered separately due to their homogeneous clinical presentation (hematuria, crystalluria or urolithiasis).
- Acute/subacute inflammation: samples from dogs that at the time of sampling had an acute/subacute presentation, with clinical or laboratory signs associated with acute presentations with inflammation, on which an infectious cause was suspected based on the clinical signs (e.g., fever, leukocytosis, and/or purulent exudates) or confirmed through additional testing (e.g., cytological evidence of leishmania, bacteria or fungi). This group was included in the study as a positive control group, since, based on the current information on ESR pathophysiology, an increase in the ESR is expected in samples included in this group [19].

In each group, the percentage of samples with values above the upper reference limit established for the automated MINI-PET method in our laboratory, or with an unmeasurable ESR, was recorded. Median and interquartile ranges (IQR) regarding the ESR recorded in each group were calculated and statistically compared with each other using the Kruskal–Wallis test, followed by the Scheffé test as a post hoc test for multiple comparisons. The same tests were used to assess possible differences in the age distribution among groups. Possible differences in sex distribution in groups was assessed using a Pearson chi-square analysis. Wilcoxon’s signed rank test was used to compare the results recorded in the set of acute pathogenesis groups with a primary inflammatory basis (severe/acute diseases, urinary disorders, acute/subacute inflammation) and the results recorded in the control group and in the set of chronic pathogenesis groups (mild chronic disorders, tumors, chronic kidney diseases), in which inflammation, if present, is possibly secondary to other conditions.

The total WBC counts (WBC), the neutrophil counts (PMN), and the hematocrit (Ht) of the different groups were also compared to each other using the tests described above for multiple comparison. The possible presence of correlations between the latter three hematological parameters and the ESR was assessed using a Spearman correlation test.

Statistical analysis was performed using specific software (Analyse-it v. 6.15.4, Analyse-it Ltd., Leeds, UK) working on Excel spreadsheets. The significance level was set at *p* < 0.05.

## 3. Results

### 3.1. Study Population

A total of 295 blood samples from 226 dogs referred at the Veterinary Teaching Hospital of the University of Milan were included. The demographic information on the study population enrolled in each group is shown in Table 1. Dogs with urinary tract disorders were significantly older than control dogs (*p* = 0.032). No other significant differences in the age of dogs belonging to the different groups were recorded. No significant differences were found based on sex distribution among groups (*p* = 0.110). 

Details of the study population, in terms of the sample size in each group, are shown as a Supplementary Table S1. There was a prevalence of samples included in the tumors group, followed by the groups of controls, severe/acute, and mild chronic disorders.

In some cases, more than one sample was collected from the same dog, as reported in the Section 2. For example, three dogs initially belonging to the severe/acute disease group for showing gastrointestinal signs (n = 2) and neurological signs (n = 1) were sampled either at first presentation, or after treatment, when clinical and laboratory findings were normal, and therefore the samples collected after recovery were included in the control group. The same occurred for two dogs with tumors, whose samples were included in the tumors group at first presentation, or in the control group when collected during periodical follow-up visits after the end of treatment protocols.

### 3.2. ESR Results

In 12 samples out of 295 (4.1%), the ESR was not measurable (error message—ERR—on the display). In all these 12 cases, the ERR message was confirmed by a second analysis of the blood sample. Five of these dogs belonged to the severe/acute disease group, presenting with hemorrhagic diarrhea (3), trauma (1), and foreign body (1), respectively. Three dogs were suffering from heart disease (1), dermatopathy (1), and gammopathy (1), respectively. Three samples were from the tumor group, including one collected during the follow-up of a dog with carcinoma and two samples collected during the follow-up of a dog with lymphoma. Finally, only one dog in the acute/subacute inflammation group with odontolithiasis and periodontitis had this result. In 9/12 cases, the unmeasurable samples were characterized by low Ht. In 5/12 cases icteric plasma was also detected.

The results recorded in the remaining 283 cases, grouped as described above, are shown in Table 2. 

**Figure 1:**
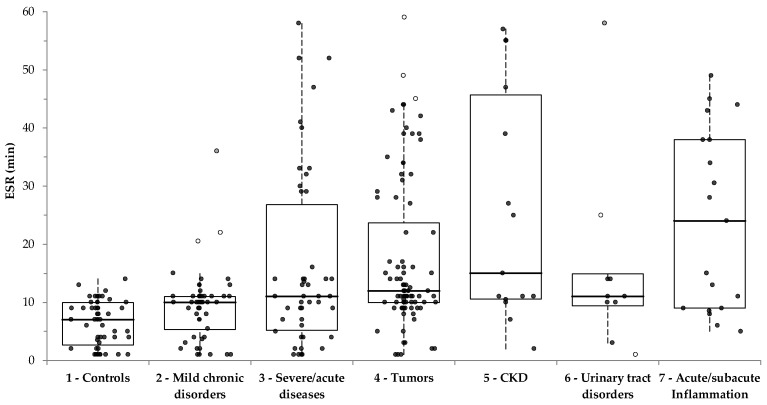
Distribution of results regarding the erythrocyte sedimentation rate (ESR) in the different groups of samples. The boxes indicate the I-III interquartile range (IQR), the horizontal black line indicates the median values, and the whiskers extend to further observation within quartile I minus 1.5 × IQR or to further observation within quartile III plus 1.5 × IQR. Black dots indicate the results that are not classified as outliers. White dots indicate near outliers (values exceeding the third quartile ± [1.5 × IQR]) and grey dots indicate far outliers (values exceeding the third quartile ± [3.0 × IQR]).

The ESR was higher than the URL in 70/295 samples (23.7%). Excluding the controls, this percentage increased to 28.9% (70/242 samples). The ESR was significantly higher (*p* = 0.039) in the set of groups in which inflammation was likely primary (severe/acute diseases, urinary disorders, acute/subacute inflammation) (median: 13.0, IQR: 7.2–30.4) compared to controls and the set of groups in which inflammation, if present, is possibly secondary to other conditions (mild chronic disorders, tumors, chronic kidney diseases) (median: 13.0, IQR: 7.0–13.0). When groups were considered separately, the highest measurable ESR values were found in the samples from the groups CKD and acute/subacute inflammation. These are also the two groups with the highest frequency of above-URL results (including ERR values), followed by the mild chronic disorders, severe/acute diseases, tumors, and urinary disorders groups. However, in all groups, the values were highly dispersed (Figure 1). For example, elevated ESR values were found only in some dogs with acute clinical presentation, that had a possible systemic involvement or inflammatory pathogenesis (trauma, hemorrhagic diarrhea, neurological symptoms), while in others, with less systemic involvement or with symptoms so acute so as to lead to the clinical examination before the onset of significant pathophysiological changes (e.g., vomiting, ingestion of foreign bodies), the ESR was normal. Similarly, in the urinary disorders group, only dogs with clinically evident cystitis showed a high ESR. On the other hand, it should be noted that the majority of dogs with elevated ESR in the tumor group were undergoing chemotherapy treatment. No dogs in the control group showed values above the URL. The ESR was significantly higher than in controls in all the pathologic groups, except than in the groups of mild chronic disorders and urinary disorders.

### 3.3. Hematological Parameters and ESR

Except for the groups with mild chronic disorders and urinary disorders, significantly lower median hematocrit values were recorded in all the disease groups when compared with controls; the lowest median hematocrit values were recorded in the CKD and acute/subacute inflammation groups (Table 3). 

WBC and PMN counts were statistically higher in samples belonging to the severe/acute diseases groups when compared with controls and with tumors. The IQRs show, however, that high values of WBC and PMN were also occasionally found in all the other groups. 

**Figure 2:**
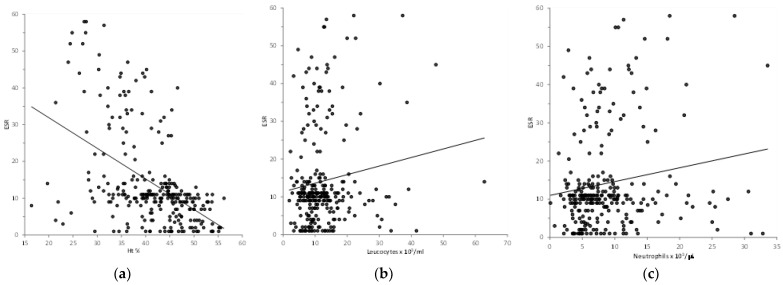
Spearman correlation between the erythrocyte sedimentation rate (ESR) and hematocrit (**a**), leucocyte count (**b**) and neutrophil count (**c**).

In general, the ESR negatively correlated with Ht (*p* < 0.001, rs = −0.496, 95% CI: −0.582 to −0.398) and positively with PMN (*p* = 0.041, rs= 0.124, 95% CI: 0.002 to 0.242), but not with WBC (*p* = 0.280, rs = −0.065, 95% CI: −0.058 to 0.185) (Figure 2).

## 4. Discussion

Although the erythrocyte sedimentation rate (ESR) is considered a marker of inflammation [19], anecdotal findings from our laboratory suggest that the ESR can also increase in clinical conditions other than inflammation, probably because of diseases that, despite not being primarily inflammatory, may secondarily induce a subclinical inflammation [31,32,33,34]. To assess the consistency of such anecdotal information and to provide reliable information to be used in clinical practice, we designed this study with the aim of identifying the clinical conditions that may alter the ESR, and to investigate the possible association between an increased ESR and changes in other routinely tested hematological parameters. The latter could be a preliminary approach to designing future studies focused on the possible pathogenic mechanism of this finding in the clinical conditions in which the ESR may frequently increase.

For this purpose, the ESR was measured in randomly selected samples sent to the laboratory from the veterinary hospital. Samples were grouped according to medical history, clinical presentation, physical examination, and the results of the diagnostic procedures.

This, however, led to the formation of groups that partially overlapped with each other in terms of pathogenetic events potentially responsible for the increased ESR. Nevertheless, this approach allowed us to clearly define the two groups that should serve as negative and positive controls, respectively, i.e., the control group and the acute/subacute inflammation group, and to compare the results of other groups, formed according to the rationale described in the Section 2, to assess whether increases in the ESR are present even in the absence of a clinically evident inflammatory event. 

Dogs were included in the former groups irrespective of previous or current treatment, since the aim of the study was to assess the possible association between ESR values and the presence of clinical changes in routine practice and not to assess the effect of a specific treatment on the ESR. From this standpoint, it cannot be excluded that samples at admission were collected from dogs receiving treatments administered or prescribed before the first presentation at our hospital. This is particularly true for dogs with chronic conditions that were mostly referred to our hospital for second opinions, often after unsuccessful or poorly successful treatments. On one hand, the possible treatments may have induced the decrease in the ESR, including in samples from dogs belonging to groups in which the ESR is expected to increase. On the other hand, this possible influence further simulates what routinely happens in clinical practice, where previous treatments are sometimes not known or not communicated by the owners. Moreover, as described in the Section 3, for a few dogs, the follow-up included visits performed after treatment and therefore some samples were allocated in the different groups based on clinical and laboratory results obtained at the end of treatment, if the clinical condition improved. Conversely, dogs undergoing chemotherapy were retained in the tumor group regardless of the tumor treated, the drug administered, or the course of treatment, because reports from human medicine suggest that the ESR may also reflect host–cancer interactions during treatments [35,36,37], and, in turn, the ESR in treated patients may be indirectly affected by the changes in Ht values induced by chemotherapy [38]. However, this aspect has not been fully investigated in veterinary medicine. 

Apart from these considerations on the rationale used to group the samples, in almost all the groups, samples with abnormal ESR results compared with the reference intervals (RI) were present. In this regard, it should be noted that the upper reference limit (URL) used in this study (ESR = 14 mm/h) is higher than the one recommended by the manufacturer of the instrument (ESR = 10 mm/h) and those used in previous studies [19,26]. This approach was based on the routine results recorded in our laboratory, that in clinically healthy dogs are often slightly higher than the recommended URL, as also occurred in the control group of this study. Therefore, we used the RI established in our laboratory according to the guidelines recommended by the ASVCP [39], corresponding to 1.0–13.7 (95% confidence interval of the URL: 13.0–14.0) using the Reference Value Advisor macroinstructions (version 2.1) for Excel (Microsoft Corp., Redmond, WA, USA) [40].

Using this RI, samples with an ESR higher than the control group or samples that provided the ERR message were found in all the pathologic groups. We can assume that all these samples had a very high ESR value, since in a previous study performed with the same instrument all the samples displaying the ERR message were characterized by a very high ESR when measured with the conventional Westergren method [26]. Nevertheless, it cannot be excluded that the ERR message depends on other factors that may interfere with the ability of the instrument to detect a clear line or separation between the settling of erythrocytes and the plasma, such as the presence of reticulocytes or of abnormally shaped erythrocytes. For example, in our caseload, the ERR message was often associated with the presence of icteric plasma, and this may have possibly contributed to masking the interface between cells and plasma. In all the other cases the instrument was able to generate the ESR result. In these cases, except for controls, the values were very dispersed around the median value in all the groups. This wide distribution probably reflects the heterogeneous composition of the groups in terms of the underlying disease, clinical presentation, and possible effects of previous treatments, as specified in the Section 3. 

However, although outliers were found in all groups, probably because of the above heterogeneity, when the samples were dichotomized according to the presence or absence of primary inflammatory events, high or very high values were more frequent in the group of subjects with primary inflammatory conditions than in the group that included dogs in which inflammation, if present, was probably mild or secondary to other chronic diseases. Similarly, when the results of the different groups were considered separately, high ESR values were found in the groups of acute/subacute inflammation, as expected based on the pathophysiology of ESR [19] and in those with CKD, as already reported in veterinary literature. The latter finding could depend on the possible presence of inflammation, which occurs secondary to CKD [34] or intrinsically in association with it, in line with recent evidence on markers of kidney damage, which redefined “chronic” progression of CKD rather as the perpetuation of active, ongoing kidney damage [41]. Nevertheless, total leukocytes and neutrophils were not significantly higher in samples with CKD than in controls. This suggests that the ESR may increase earlier than leukocytes, but this should apply to acute-onset diseases rather than to CKD, which is chronic by definition. Therefore, the increased ESR in the CKD group may depend on the decrease in Ht that may characterize this condition. The inverse relationship between ESR and Ht is well known [23,28], and in the current study, the results of correlation tests performed on the whole caseload confirmed that the presence and magnitude of anemia may have induced increases in the ESR, both in dogs with acute inflammation and in dogs with CKD. The Ht, however, was significantly lower than in controls, as well as in other groups characterized by an increased ESR, especially in samples of the tumor group, which was also characterized by a moderately high prevalence of ESR values higher than the RI.

The other hematologic variable that was positively correlated with ESR in the entire case series was the neutrophil count, which, as expected, was elevated in dogs with acute symptomatology. Although this finding is likely secondary to the response toward inflammatory stimuli, a stress leukocytosis cannot be ruled out in these subjects through the release of cells from the marginal pool [42]. Neutrophil counts were particularly high, although not significantly, in samples from dogs with acute/subacute inflammation as a direct consequence of the activation of the acute phase reaction induced by the inflammatory stimuli [43]. In other groups with a high ESR, leukocyte and neutrophil counts were not elevated, suggesting that the inflammatory trigger in these groups is not particularly intense or that, as stated above, the increase in the ESR may occur earlier than leukocytosis. This latter hypothesis, however, is not supported by the results of studies in humans, which did not show an earlier increase in the ESR compared with leukocyte counts [44,45]. Moreover, among dogs with tumors, the absence of evident neutrophilia can be justified by the fact that most samples of this group were from dogs undergoing chemotherapy which, as a side effect, can induce neutropenia, masking any inflammatory responses. However, the design of this study, and in particular the heterogeneous composition of the tumor group, either in terms of the type of tumor or in terms of the type, dosage, and frequency of administration of chemotherapeutic drugs, does not allow us to understand whether the increase in the ESR recorded in samples from dogs undergoing chemotherapy independent of Ht or WBC/neutrophil changes may be a direct consequence of treatments. This aspect deserves to be further explored in future studies on a more homogeneous caseload, possibly investigating the ESR in each type of tumor and/or with a more standardized protocol of sampling during treatments.

This study has several limitations, such as the heterogeneous composition of groups, the possible coexistence and overlap of multiple pathogenic mechanisms in some of the samples or groups, and the lack of standardization of type, duration, and severity of the disease or treatments, which have already been discussed. However, all these aspects are intrinsically present in any preliminary field study and ultimately allow us on one hand to increase the caseload, and on the other to better reflect the routine clinical activity compared with smaller studies on highly selected and standardized samples. These latter studies can now be performed on dogs with CKD and tumors, that, and among the diseases that do not have a primary inflammatory component, are those on which ESR was more frequently increased in the current study. These future studies should also take into account the other details of the caseload that have not been considered here, such as the actual presence of other inflammatory changes through the measurement of inflammatory markers, such as acute phase proteins, that have not been consistently determined in the current caseload and therefore not considered in data analysis, or of other variables potentially able to influence the severity of the disease (e.g., IRIS stage in the CKD group).

## 5. Conclusions

An increased ESR occurs most frequently in dogs with clinically evident inflammation or in the course of CKD, and is associated with increased leukocyte counts and/or decreased Ht, as previously reported [19]. Based on the current results, increases in the ESR can also be seen in conditions other than those mentioned above. Dogs with tumors show an increase in the ESR. Having included subjects with different malignancies in this group, either before or during the chemotherapy treatment, further studies are needed to clarify whether tumor type and ongoing chemotherapy may affect the ESR.

## Figures and Tables

**Table 1:** Breed, sex, and age of the dogs included in the different groups. Age is reported as median value and range (between parentheses).

Group	N	Breed	Sex	Age
Controls	53	Crossbred (n = 13), Breed unknown (n = 7), Labrador retriever (n = 6), Chihuahua (n = 4), German shepherd (n = 3), Belgian shepherd, Cavalier King Charles spaniel, Bolognese, Poodle (n = 2), American bulldog, Dachshund, Beagle, Entlebucher mountain dog, Drahthaar, Pug, Swiss mountain dog, Jack Russell terrier, Kurzhaar, Abruzzese shepherd, Bergamasco shepherd, German pinscher (n = 1)	17 F 8 Fs 24 M 2 Mc 2 Unk	6.3 (0.4–16.6) 6 Unk
Mild chronic disorders	53	Crossbred (n = 9), Chihuahua (n = 6), Yorkshire terrier (n = 5), Breed unknown, German shepherd (n = 4), Golden retriever (n = 3), Poodle, Dachshund, Cavalier King Charles spaniel, English setter, French bouledogue (n = 2), Italian bracco, Chow chow, Corso, Dobermann pinscher, English Staffordshire terrier, Jack Russell terrier, Labrador retriever, Maltese, Pitbull terrier, Toy schnauzer, Shi tzu (n = 1)	11 F 16 Fs 24 M 1 Mc 1 Unk	9.3 (0.4–15.3) 4 Unk
Severe/acute diseases	52	Breed unknown (n = 11), Crossbred (n = 9), Golden retriever (n = 5), Dobermann pinscher (n = 4), American Staffordshire, (n = 3), Cavalier King Charles spaniel, Jack Russell terrier, Italian segugio, French bouledogue (n = 2), Australian shepherd, Dobermann pinscher, Dogo argentino, Drahthaar, Labrador retriever, German shepherd, German Pinscher, Pitbull terrier, Pointer, Rottweiler, Shar pei, Shiba inu, Whippet (n = 1)	11 F 18 Fs 19 M 3 Mc 1 Unk	8.0 (0.4–16.0) 7 Unk
Tumors	92	Crossbred (n = 14), Rottweiler, English setter (n = 10), Golden retriever (n = 8), German shepherd (n = 6), Boxer (n = 5), Beagle, French bouledogue, West highland white terrier (n = 4), Australian shepherd, Poodle (n = 3), Breed unknown, Bull terrier, Dogue de Bordeaux, German pinscher, Pitbull terrier (n = 2), Dachshund, Bernese mountain dog, Cavalier King Charles spaniel, Jack Russell terrier, Labrador retriever, Afghan sighthound, Abruzzese shepherd, Bergamasco shepherd, Tosa inu, Welsh Corgi Pembroke, Yorkshire terrier (n = 1)	22 F 25 Fs 30 M 14 Mc 1 Unk	9.4 (1.9–14.5) 8 Unk
CKD	15	Crossbred (n = 4); Boxer (n = 3), Shi-tzu (n = 2), Italian bracco, English bulldog, Cocker, Dogue de Bordeaux, Shiba inu, Pomeranian (n = 1)	4 Fs 9 M 2 Mc	8.1 (1.8–16.6) 0 Unk
Urinary disorders	10	Crossbred (n = 2), Dachshund, Bolognese, Bull terrier, Epagneul breton, Fox terrier, Golden retriever, German pinscher, Yorkshire terrier (n = 1)	2 F 3 Fs 3 M 2 Mc	12.8 (5.1–18.6) 2 Unk
Acute/subacute inflammation	20	Crossbred (n = 5), Unknown breed (n = 3), Rottweiler (n = 2), Poodle, Basset-hound, Cocker spaniel, Setter gordon, Labrador retriever, Belgian shepherd, German shepherd, Pekingese, Italian segugio, English setter (n = 1)	10 F 2 Fs 7 M 1 Mc	6.1 (0.9–14.2) 2 Unk

F = female; Fs = spayed female; M = male; Mc = castrated male; Unk = unknown.

**Table 2:** Results regarding the erythrocyte sedimentation rate (ESR, expressed in mm/hour) recorded in the different groups. The number and percentage of samples with ESR results higher than the upper reference limit (URL) of the ESR (14 mm/hour) and the number of cases with results higher than the URL or displaying an “ERROR” message are also reported. Superscripts indicate the groups that are significantly different, along with the corresponding level of statistical significance.

Group	Median (I–III IQR)	>URL	>URL + ERR
1—Controls	7.0 (2.7–10.0) ^4,5,7 (*p* < 0.001); 3 (*p* = 0.016)^	0/53 (0.0%)	0/53 (0.0%)
2—Mild chronic disorders	10.0 (5.4–11.0) ^5,7 (*p* = 0.003); 4 (*p* = 0.039)^	4/50 (8.0%)	7/53 (13.2%)
3—Severe/acute diseases	11.0 (5.2–26.8) ^1 (*p* = 0.016)^	13/47 (27.7%)	20/52 (38.5%)
4—Tumors	12.0 (10.0–23.7) ^1 (*p* < 0.001), 2 (*p* = 0.039)^	32/89 (40.0%)	35/92 (38.0%)
5—Chronic Kidney Disease (CKD)	15.0 (10.6–45.7) ^1 (*p* < 0.001); 2 (*p* = 0.003)^	8/15 (53.3%)	8/15 (53.3%)
6—Urinary tract disorders	11.0 (9.4–14.9)	2/10 (20.0%)	2/10 (20.0%)
7—Acute/subacute inflammation	24.0 (9.0–38.0) ^1 (*p* < 0.001); 2 (*p* = 0.003)^	11/19 (57.8%)	12/20 (60.0%)

IQR = interquartile range.

**Table 3:** Hematological variables recorded in the different groups. Results are expressed as median values and I-III interquartile (IQR) between parenthesis. Superscripts indicate the groups that are significantly different, along with the corresponding level of statistical significance.

Group	Ht (%)	WBC × 10^3^/μL	Neutrophils × 10^3^/μL
1—Controls	45.0 (41.3–49.9) ^3 (*p* = 0.018); 4 (*p* < 0.001); 5 (*p* = 0.042); 7 (*p* = 0.024)^	9.8 (8.1–13.3) ^3 (*p* = 0.018)^	5.9 (4.7–8.2) ^3 (*p* < 0.001)^
2—Mild chronic disorders	44.9 (37.7–50.6) ^4 (*p* = 0.017)^	10.5 (8.2–13.8)	7.3 (5.3–10.4)
3—Severe/acute diseases	43.4 (32.4–47.0) ^1 (*p* = 0.018)^	14.8 (10.3–21.6) ^1 (*p* = 0.018); 4 (*p* < 0.001)^	11.5 (7.2–17.5) ^1 (*p* < 0.001); 4 (*p* < 0.001)^
4—Tumors	39.6 (33.2–44.0) ^1 (*p* < 0.001); 2 (*p* = 0.017)^	8.6 (5.8–12.5) ^3 (*p* < 0.001)^	6.3 (3.5–9.2) ^3 (*p* < 0.001)^
5—Chronic Kidney Disease (CKD)	36.4 (35.4–41.7) ^1 (*p* = 0.042)^	9.6 (8.6–12.9)	6.2 (4.9–9.9)
6—Urinary tract disorders	45.6 (43.1–47.4)	10.4 (7.5–14.9)	6.4 (4.6–11.6)
7—Acute/subacute inflammation	37.5 (34.7–43.7) ^1 (*p* = 0.024)^	13.0 (10.5–17.0)	9.5 (7.2–15.2)
*p*	<0.001	<0.001	<0.001

## Data Availability

The data presented in this study are available on reasonable request from the corresponding authors.

## Supplementary Materials

The following supporting information can be downloaded at: https://www.mdpi.com/article/10.3390/ani14101409/s1.
