# Supplementary material for: Increased Erythrocyte Sedimentation Rate in Dogs: Frequency in Routine Clinical Practice and Association with Hematological Changes
Source: Animals (Basel). 2024 May 8;14(10):1409. doi: 10.3390/ani14101409 (PMC11117206; doi:10.3390/ani14101409)
Supplement: Supplementary file 1 [file animals-14-01409-s001.zip › animals-2935134-supplementary.pdf]

Table S1. Details of the diseases or conditions recorded in the different groups of samples.

Group	N	Subgroup	Diseases/ conditions
Controls	53	Pre-elective surgery (n=18)	Neutering (n=10); Correction of osseous anomalies (n=3); Dental surgery (n=2); Lipoma (n=2); Perineal hernia (n=1)
		Wellness visits/vaccination (n=15)	
		Follow-up of sick animals (n=8)	Leishmaniasis after treatment and negative serology (n=3) Previous GI (n=2) or CNS (n=2); Treated Addison's disease (n=1)
		Tumors after chemotherapy of surgery (n=8)	Mast cell tumor (n=4); Carcinoma (n=2); CLL (n=1) Lymphoma (n=1)
		Diagnostic imaging (n=5)	No significant diseases (n=5)
Mild chronic disorders	53	Orthopedic disorders (n=13)	
		Non-neoplastic non-inflammatory neuropathy (n=9)	Peripheral neuropathies (n=7) Idiopathic epilepsy (n=2)
		Cardiopathy (n=6)	
		Respiratory disorders (n=6)	
		Hematologic disorders (n=6)	IMTP (n=5), Non-regenerative anemia (n=1)
		Dermatopathy (n=5)	
		Metabolic disorders (n=3)	Endocrine diseases (n=2); MGUS (n=1)
		Miscellaneous (n=5)	Cystitis (n=2); Enteropathy (n=1); Otitis (n=1); Immune-mediated uveitis (n=1)
Severe/acute diseases	52	Gastrointestinal signs (n=17)	Non specific etiology (n=14); Parvovirus (n=3)
		Foreign bodies (n=8)	Ingested (n=2); Subcutaneous (n=6)
		Acute respiratory distress (n=7)	Non specific (n=5); Pulmonary oedema (n=1); Pneumothorax (n=1)
		Acute trauma (n=5)	
		Hemoabdomen (n=4)	
		Neurological signs (n=4)	Seizures (n=3); Lipothymia (n=1)
		Miscellaneous (n=4)	Acute abdomen (n=1); GDV (n=1); Heatstroke (n=1); Pesticide poisoning (n=1)
		Hematologic disorders (n=3)	IMHA (n=3)
Tumors	92	First presentation (n=45)	Carcinoma (n=14); Lymphoma (n=11); MCT (n=6); Insulinoma (n=2); Osteosarcoma (n=2); CLL (n=1); Lipoma (n=1); Melanoma (n=1); TVT (n=1); Chemodectoma (n=1); Histiocytic sarcoma (n=1); Splenic sarcoma (n=1); Oral papilloma (n=1); Sertolioma (n=1); STS (n=1)
		During treatments (n=47)	Lymphoma (n=23); Carcinoma (n=15); MCT (n=3); Insulinoma (n=2); Histiocytic sarcoma (n=1); Chemodectoma (n=1); STS (n=1); Osteosarcoma (n=1)
CKD	15		
Urinary disorders	10	Cystitis and hematuria (n=6)	
Acute/sub-acute inflammation	20	Purulent inflammation (n=14)	Pyometra (n=4); Purulent inflammation on ulcerated tumors (n=4); Skin abscesses (n=3); Purulent otitis (n=2); Purulent rhinitis (n=1)
		Leishmaniasis (n=3)	
		Miscellaneous (n=3)	Odontolithiasis (n=1); Prostatitis (n=1); Systemic mycosis (n=1)

GI = gastrointestinal; CNS = central nervous system; CLL = chronic lymphocytic leukemia; MGUS = monoclonal gammopathy of unknown significance; MCT = mast cell tumor; TVT = transmissible venereal tumor; STS = soft tissue sarcoma; CKD = chronic kidney disease; IMTP = immune-mediated thrombocytopenia; IMHA = immune-mediated hemolytic anemia.
